# A unique biofilm in human deep mycoses: fungal amyloid is bound by host serum amyloid P component

**DOI:** 10.1038/npjbiofilms.2015.9

**Published:** 2015-07-08

**Authors:** Melissa C Garcia-Sherman, Tracy Lundberg, Richard E Sobonya, Peter N Lipke, Stephen A Klotz

**Affiliations:** 1 Department of Biology, City University of New York, Brooklyn College, Brooklyn, NY, USA; 2 Department of Pathology, University of Arizona, Tucson, AZ, USA; 3 Department of Medicine, University of Arizona, Tucson, AZ, USA

## Abstract

**Background/objectives:**

We have demonstrated the presence of *Candida* cell surface amyloids that are important in aggregation of fungi and adherence to tissue. Fungal amyloid was present in invasive human candidal infections and host serum amyloid P component (SAP) bound to the fungal amyloid. SAP is a protease-resistant glycoprotein that binds avidly to amyloid and interferes with host defence, especially against bacterial pathogens for which neutrophils are important. In this study, we investigated whether biofilm of fungal amyloid and SAP was a feature of other disseminated fungal infections.

**Methods:**

Tissue specimens from 15 autopsies were systematically evaluated with multiple histochemical stains including thioflavin T and Congo red (dyes that stain amyloid), as well as antibody to SAP. We studied specimens with disseminated aspergillosis, mucormycosis and coccidioidomycosis. The structure of the lesions, host inflammatory cells and the presence of fungal amyloid and SAP were determined.

**Results:**

The structure of the lesions was characteristic in aspergillosis (‘starburst’) and mucormycosis (closely apposed bundles of hyphae). Host inflammatory cells were absent or few in number within these lesions. In *Coccidioides* lesions, host inflammation was sparse as well. Fungal amyloid was a prominent feature of all lesions along with abundant SAP bound to hyphae and spherules. Fungal amyloid and SAP perhaps contributed to persistence in caseous necrosis lesions. SAP also bound to *Aspergillus* and Mucorales amyloid *in vitro*.

**Conclusions:**

A biofilm including amyloid and SAP is present in invasive fungal infections. This biofilm may dampen host defence leading to the characteristic sparse inflammatory reaction found in these infections.

## Introduction

In human deep mycoses such as invasive pulmonary aspergillosis and pulmonary mucormycosis, fungi serve as a substratum for a biofilm within invaded tissue that often has little or no cellular inflammatory response. This characteristic feature is usually attributed to a lack of production of neutrophils often due to use of cytoxic drugs.^1^ However, systematic investigation of these two mycoses does not support this statement. Invasive pulmonary aspergillosis patients with neutropenia or normal white blood cell counts have the same histopathological pattern, that is, invasive fungi with an absence of host inflammation.^2^ This finding also characterises pulmonary mucormycosis.^3^ Therefore, other causes may account for the absent or weak inflammatory infiltrate in these diseases.

We showed in invasive candidiasis of the gastrointestinal tract that fungal cell surfaces were coated with amyloid along with host serum amyloid P component (SAP) and as a consequence, the inflammatory response was scant or absent.^4^ Here we show in the deep mycoses of aspergillosis, mucormycoses and coccidioidomycosis an abundance of fungal amyloid coated with SAP. The amyloid and SAP form a protease-resistant biofilm^5^ that contributes to the stereotypical histology of the lesions with little or no inflammatory reaction. Amyloid and SAP, an invariant component of amyloid in humans,^6^ characteristically do not elicit an inflammatory response. The presence of fungal surface amyloids and the subsequent binding of SAP may diminish the inflammatory response needed for clearance of infection.

## Patients and Methods

### Tissue stains

Autopsy specimens from 15 patients with histological evidence of invasive aspergillosis (three patients), mucormycosis (three patients) and coccidioidomycosis (nine patients) were examined. Tissue blocks were cut 4 μ thick, deparaffinised and stained with haemotoxylin and eosin (H&E) and Gomori methenamine silver (GMS).^7^ Fungi were identified on H&E or GMS stains. Slides found to be positive for *Aspergillus*, Mucorales and *Coccidioides* were then stained with a polyclonal antibody to SAP (Biocare Medical, LLC; Concord, CA, USA). Congo red (0.1%) and 100 nM thioflavin T stains for amyloid^8^ were also performed on tissue specimens as previously published.^4^ Thioflavin T fluorescence images were acquired on a Nikon Eclipse 90i confocal microscope with 408 nm excitation and 450 nm emission filters (Nikon, Melville, NY, USA). Studies of thrush (oral pharyngeal candidiasis) were performed on material scraped from an AIDS patient with pseudomembranous thrush.

### Fungi

*Rhizopus nigricans* and *Aspergillus flavus* were obtained from the clinical microbiology laboratory and maintained on yeast extract peptone dextrose (Life Technologies, Grand Island, NY, USA) agar. The isolates were anonymous with respect to patient origin and did not derive from any of the autopsies. Both species were cultured in yeast extract peptone dextrose broth for 8–10 h at 37 °C, washed in tris-buffered saline, pH 7.4 and then heat-killed at 80 °C for 2 h in a water bath and washed in tris-buffered saline, pH 7.4 with 4% bovine serum albumin and 2 mM Ca^++^. Fungi were fixed on microscope slides and incubated with human serum (the source of SAP) or tris-buffered saline, pH 7.4, with 4% bovine serum albumin and 2 mM Ca^++^ for 2 h, washed with buffer and incubated with SAP antibody (Biocare Medical) for 1 h. The slides were washed again with the same buffer and fungi incubated with 1/500 Alexa Fluor 555 goat anti-rabbit IgG (Life Technologies) in tris-buffered saline with 4% bovine serum albumin and 2 mM Ca^++^ for 1 h, washed and observed with a DeltaVision deconvolution microscope (GE Healthcare, Issaquah, WA, USA). All incubations were performed at 26 °C. Both antibody preparations were pre-absorbed against the respective heat-killed fungi overnight at 26 °C.

### Insitutional Review Board (IRB)

Autopsy material is exempt from IRB oversight. The tissue in this study is not from patients and next of kin in all cases signed consent forms releasing tissues for research use. Fungi used in this study were de-identified, considered case studies and were exempt from IRB oversight.

## Results

**Figure 1:**
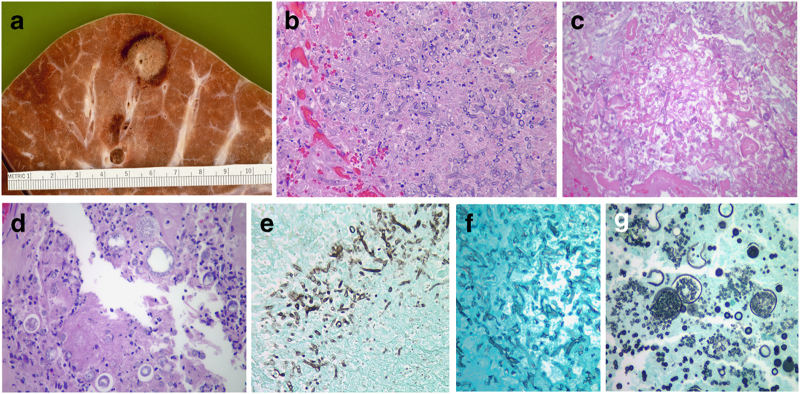
Histology of fungal aggregates *in situ*. (**a**) View at autopsy of a stereotypical ‘starburst’ lesion of invasive *Aspergillus* of the lung (tan tissue) encircled by a halo of haemorrhage. (**b**) H&E stain demonstrating *Aspergillus* hyphae (light-blue structures in the centre of the photomicrograph) radiating through lung tissue and surrounded by a collar of haemorrhage. Note the absence of inflammatory cells around and within the tangle of fungal hyphae (×400). Compare **b** with **e** below it where darkly stained hyphae stand out from the acellular background. Hyphae are ~7 μ in diameter. (**c**) H&E stain of heart tissue with a myriad of ribbon-like, non-septate hyphae of a diameter of ~10 μ occupying the majority of the photomicrograph. These features are diagnostic of Mucorales species. Note the absence of host inflammatory cells within and surrounding the hyphal bundles. (**d**) H&E stain of lung tissue of a patient with pulmonary coccidioidomycosis demonstrating various sizes of spherules (20–50 μ) with and without endospores surrounded by a sparse inflammatory infiltrate (×400). (**e**) GMS of lung tissue showing *Aspergillus* hyphae (black structures) radiating outwards from a central focus or ‘starburst’ (×400). (**f**) GMS stain of heart showing large, black, non-septate hyphae. Note the density of the hyphae in the lesion. (**g**) GMS stain of lung tissue showing numerous spherules (black circles) and endospores (black dots); some spherules are empty and do not contain endospores, others are broken open and ‘spilling’ endospores (×400).

**Figure 2:**
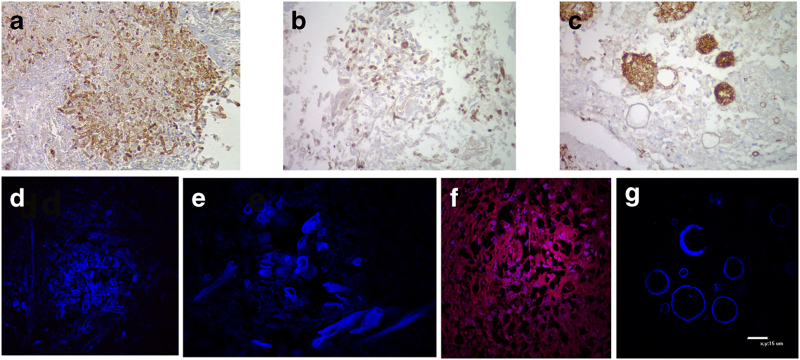
SAP and thioflavin T binding to fungal aggregates *in situ*. (**a**) Lung tissue stained with antibody to SAP that is bound to the wall of the *Aspergillus* hyphae (SAP stains a reddish-brown). This illustrates the spherical ‘starburst’ nature of the lesion (×400). (**b**) Heart tissue stained with antibody to SAP demonstrating patchy staining, often at junctions of the hyphae of Mucorales (×400). (**c**) Lung tissue stained with antibody to SAP showing heavy staining (reddish-brown) of endospores and spherule walls of *Coccidioides* (×400). (**d**) Thioflavin T stain of lung tissue containing hyphal elements of *Aspergillus* that are fluorescent and arranged in a ‘starbust;’ some hyphae are cut in cross-section and appear as tubular forms. The fluorescent structure within the hyphae is amyloid. (**e**) Thioflavin T stain of brain tissue demonstrating ribbon-like hyphae of Murcorales in oblique and cross-section. (**f**) Lung tissue stained with Congo red and thioflavin T demonstrating fluorescent endospores (numerous blue–red structures) originating from a spherule and scattered throughout lung tissue (stained red with Congo red). (**g**) Splenic tissue from a patient with disseminated coccidioidomycosis infiltrated with spherules that fluoresce with thioflavin T, demonstrating the amyloid in the spherule walls (×400).

Three autopsy cases with disseminated aspergillosis were studied. Tissue from brain, lung and kidney were stained. *Aspergillus* was cultured from each case at autopsy (Supplementary Table 1). A stereotypical lesion of this disease is shown in Figure 1a with a spherical infiltrate surrounded by faint haemorrhage located in the periphery of the lung. Fungi are readily identified microscopically in H&E- and GMS-stained tissue (Figure 1b,e) and in all cases appeared as bundles of closely apposed hyphae, the advancing edge radiating outwards in a ‘starburst’ arrangement (suggested in Figures 1a,e and 2a). Within fungal bundles there was little or no identifiable host cellular inflammatory response (appreciated on H&E, Figure 1b). Two of three of the autopsy cases showed deposition of SAP on and within the cell walls of the hyphae in lung and brain tissue (Figure 2a). One specimen from an invasive lesion of the kidney did not show evidence of SAP association with the fungi, but it had undergone autolysis. It should be noted that fixation in formalin and embedding tissue in wax (as was done with these specimens) can damage the immunoreactivity of SAP,^9^ and this may have affected the results. Thioflavin T staining demonstrated amyloid throughout the length and breadth of all fungi in tissue (Figure 2d). Many of the fungi are cut in cross-section. Some, however, were captured in oblique section and it is apparent that amyloid is present throughout the fungal cell wall, not only on the hyphal surface.

Three autopsy cases with invasive Mucorales infections were studied (Supplementary Table 1). Tissue included brain, heart, larynx and lung. Fungus was cultured from one autopsy. Pathognomonic ribbon-like, non-septate hyhae were readily visible on H&E- and GMS-stained tissue (Figure 1c,f). Host cells were entirely lacking within the bundle of hyphae seen on the H&E stain, a finding very similar to that seen with *Aspergillus.* The Mucorales infections differed from aspergillosis, in that rather than a ‘starburst’ the hyphae were bundled together often travelling in the same direction. Two of the autopsy cases had evidence of deposition of SAP onto the fungal surface in tissue from the brain, heart and larynx (Supplementary Table 1; Figure 2b) often at the junction of branching hyphae. Hyphae stained uniformly with thioflavin T, demonstrating distribution of functional amyloid throughout the hyphal walls (Figure 2e) similar to *Aspergillus*.

**Figure 3:**
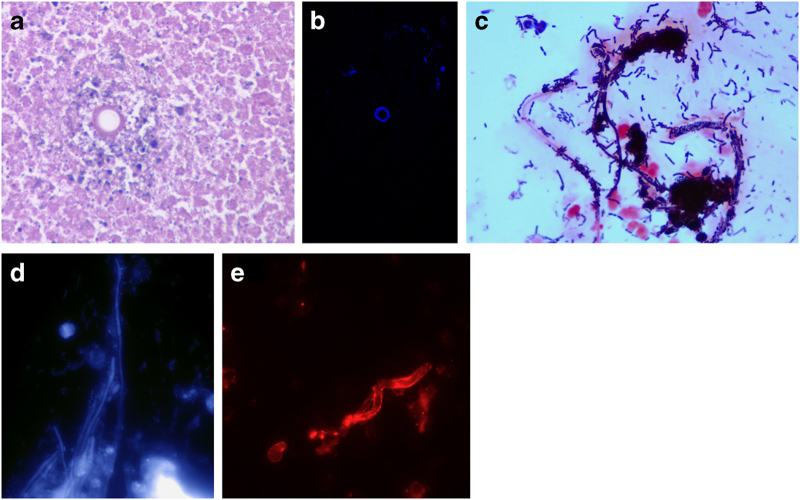
SAP binding *in situ* and *in vitro*. (**a**) Lung tissue with a *Coccidioides* spherule in a granuloma undergoing caseous necrosis. The spherule is surrounded by eosinophilic cellular debris (H&E, ×400). (**b**) Same spherule stained with thioflavin T demonstrates the amyloid present in this empty, likely non-viable spherule that is impervious to degradation by the caseous process. (**c**) Gram stain of scraping from oropharyngeal thrush showing hyphae, yeast cells and Gram-positive bacteria adherent to and encasing the fungi with a paucity of host cells. (**d**) Thioflavin T stain of scraping from the same patient demonstrating amyloid in hyphae or pseudohyphae of *Candida*. (**e**) Condidia of *Aspergillus flavus* were cultured for 8 h in yeast extract peptone dextrose, washed and incubated in human serum for 2 h, followed by washing and staining for SAP with fluorescent antibody (red). Note: SAP decorated the entire hyphal surface.

Spherules in tissue are pathognomonic of infection with *Coccidioides* species. Nine autopsy specimens demonstrated spherules in the lung; three cases had evidence of dissemination to other tissue including the brain, spleen, peritoneum and thyroid tissue. Individual spherules of various sizes and maturation were visible on H&E with few neutrophils present in the tissue (Figure 1d). GMS stain (Figure 1g) showed numerous spherules, some ‘spilling’ endospores. A sparse inflammatory response was present and there were few neutrophils. Deposition of SAP was strongest on endospores within spherules, as well as the cell wall of spherules (Figure 2c). Staining with Congo red and thioflavin T demonstrated fluorescent endospores (Figure 2f) scattered within lung tissue. In addition, thioflavin T staining showed empty spherules composed of amyloid throughout the width of the spherule wall with strongly stained material around the inner circumference of the cell wall (Figure 2g). One specimen demonstrated the durability of fungal amyloid in caseous necrosis. Caseous necrosis is a host response mounted in an attempt to ‘wall off’ a pathogen. As seen in Figure 3a, all host tissue was autodigested leaving only spherules with amyloid-impregnated cell walls (Figure 3b). The surrounding material is host cellular debris leaving a caseous or ‘cheesy’ deposit. These lesions may be present in patients for years.

Thrush is a superficial mucosal infection caused by *Candida* species, and we looked for the expression of amyloid in this disease. Scraping pseudomembranous lesions of the oropharynx yielded curds of material coated with saliva. Gram stain of curds showed a mixed biofilm of numerous filamentous and yeast forms of the fungus, each heavily decorated with oral bacteria (Figure 3c). Staining curds with thioflavin T demonstrated extensive amyloid in the hyphae, as well as within some adherent bacteria (Figure 3d). Fungi in this lesion were arranged in a bundle-like scaffold, adjacent to one another, and at some points there was apposition of the cell surfaces. There were few host cells found in this lesion. Fungal amyloid appeared to contribute to the cellular architecture of this macroscopic, amorphous biofilm-like lesion.

*Aspergillus* and *Rhizopus* (a Mucorales) were studied *in vitro* to determine whether SAP bound to their surfaces as it does in tissue. SAP bound to the surface of both fungal hyphae (Figure 3e and data not shown) as well as to the surface of the conidiospores of *Aspergillus* and the sporangiospores of *Rhizopus*. SAP decorated entire hyphal surfaces similar to what occurred *in vivo*.

## Discussion

Extracellular functional amyloids are common among microbes, fastening them to substrates such as host cells and other microbes,^10–13^ as well as contributing to the acellular composition of the biofilm.^14^ Because these amyloids perform a beneficial function for the microbe, they are referred to as ‘functional amyloids’ in contrast to terms such as amyloidosis and amyloid deposits, which signify pathological aggregates of proteins in tissue or cells. Functional amyloids are believed to be important in the pathogenesis of some infectious diseases, for example, curli protein of *Escherichia coli*,^13^ pili of *Mycobacterium tuberculosis*^15^ and merozoite surface protein 2 of *Plasmodium falciparum.*^16^ These functional amyloids attach their respective microbes to host tissue.

Demonstrating the presence of microbial functional amyloids in human disease is difficult, in part because of their small size as well as lack of knowledge concerning their composition and function. In our studies with candidiasis amyloid was detected on hyphae and yeast cells where it forms spontaneously as aggregates of cell surface adhesion molecules.^17,18^ The Als adhesins of *Candida albicans* attach fungi to host tissue and other *Candida* cells,^19^ are demonstrable in infected tissue^4,20^ and are antigenic.^21^ Such functional amyloids are common in fungal adhesins.^22^ We hypothesised that SAP bound to the fungal amyloid and masked fungi from the host defence in spite of the antigenicity of the proteins.

Functional amyloids could contribute physically to excluding host cells due to their extremely tight fungal cell–cell adhesion mediated by amyloid-forming adhesins. Fungal hyphae radiate out from a central focus in aspergillosis (‘starburst’) and in bundles, in the case of Mucorales. These fungi are considerably larger than host cells, and their close apposition at their margins poses a physical barrier to penetration by inflammatory cells. In aspergillosis, a ‘starburst’ of hyphae grows from a central point and is surrounded by coagulation necrosis, which in turn is surrounded with haemorrhage (Figure 1a). The caseous tissue with spherules provides testimony for the persistence of amyloid in tissue (Figure 3b) long after the loss of viability of the fungus.

SAP binds to all known amyloid fibrils and is invariably present where amyloid is found in humans.^6^ In amyloidoses, SAP constitutes about 15% of the amyloid deposits leading to tissue and organ dysfunction.^23^ SAP covers amyloid fibrils and masks host recognition of the abnormal protein (amyloid) and protects the amyloid from degradation by the host.^5^ Amyloid bound by SAP is characterised by a total absence of an inflammatory response. SAP also binds to bacteria and has a powerful anti-opsonic effect interfering bacterial phagocytosis by neutrophils.^24^

Fungal amyloid and SAP in these deep mycoses may be responsible for the exiguous inflammatory response seen in invasive candidiasis,^4^ as well with infections including *Aspergillus*, Mucorales and *Coccidioides* as shown here. This discovery may have opened a new chapter in the pathogenesis of invasive fungal diseases as well as novel therapies.^25^
