# Supplementary material for: A unique biofilm in human deep mycoses: fungal amyloid is bound by host serum amyloid P component
Source: NPJ Biofilms Microbiomes. 2015 Jul 8;1:15009–. doi: 10.1038/npjbiofilms.2015.9 (PMC4563996; doi:10.1038/npjbiofilms.2015.9)
Supplement: Supplementary Information [file npjbiofilms20159-s1.doc]

| **Microorganism** | **Autopsy Number** | **Tissue Infected** | **SAP results** | **Comments** |
| --- | --- | --- | --- | --- |
| *Aspergillus* spp*.* | 1 | kidney | Negative (tissue was autolysed) | *Aspergillus* species on culture; white blood cell count of 27,300/μL |
|  | 2 | lung | positive | *Aspergillus* species on culture; acute lymphocytic leukemia |
|  | 3 | brain | positive | *Aspergillus* species on culture; acute lymphocytic leukemia |
| Mucorales | 4 | brain | patchy, weak | *Staphylococcus* osteomyelitis; intravenous drug use |
|  | 5 | heart, larynx | patchy, weak | Bone marrow transplant |
|  | 6 | lung | negative | *Rhizopus* species on culture; white blood cell count, 1,200/uL; bone marrow transplant |
| *Coccidioides* | 7 | lung | weakly positive | No immune suppression in *Coccidioides* cases |
|  | 8 | lung | weakly positive |  |
|  | 9 | lung | weakly positive |  |
|  | 10 | lung | weakly positive |  |
|  | 11 | lung | weakly positive |  |
|  | 12 | brain | positive |  |
|  | 13 | spleen lung, peritoneum | positive |  |
|  | 14 | lung, spleen, thyroid | positive |  |
|  | 15 | lung, caseous necrosis | positive |  |

**Table 1. Ancillary data on 15 autopsies of deep mycoses.**
